# C-855-01. A Multicenter Framework for Frostbite Care: Findings from the FROST Study

**DOI:** 10.1093/jbcr/irag033.085

**Published:** 2026-04-06

**Authors:** Lucy Wibbenmeyer, Rachel M Nygaard, Emily Colonna, Lee D Faucher, Alexander J Poole, Rosemary E Paine, Arek J Wiktor, Alexandra M Lacey, Irma D Fleming, Kristin P Colling, Naveen F Sangji, Joshua S Carson, Alexis Armour, Damien W Carter

**Affiliations:** University of Iowa Health Care, Iowa City, IA; Hennepin Healthcare, Minneapolis, MN; Hennepin Healthcare Research Institute, Minneapolis, MN; University of Wisconsin Burn and Wound Center, Madison, WI; Whitehorse General Hospital, Whitehorse, Yukon Territory; MaineHealth Medical Center, Portland, ME; Burn & Frostbite Center at UCHealth, University of Colorado Hospital, Aurora, Colorado; Regions Hospital, Saint Paul, MN; University of Utah Health Burn Center, Salt Lake City, UT; Essential Health, Duluth, MN; University of Michigan, Ann Arbor, MI; Loyola Burn Center, Maywood, IL; University of Alberta, Edmonton, AB; MaineHealth Medical Center, Portland, ME

## Abstract

**Introduction:**

Frostbite results from atmospheric cooling of tissues and poses significant morbidity risks that impact both military personnel and civilians. Lasting sequelae occur in 50–85% of cases. While amputations represent the most feared outcome, other chronic consequences include cold intolerance, pain, paresthesia, and loss of occupational function. In an attempt to develop evidence-based practice, we sought to examine frostbite treatment protocols from different institutions.

**Methods:**

The multicenter FROST study seeks to harmonize data collection of current treatments and outcomes at centers across North America. To do this we aggregated institutional protocols across 16 centers. Three reviewers independently assessed protocols, denoted the presence or absence of specific treatments, and detailed specific timing and medical management details. Summative data was calculated and tables were generated including: overall management, rewarming, therapeutic windows and dosing for pharmaceutical interventions, indications and contraindications for thrombolytics and/or iloprost, post-thrombolytic anticoagulation, rehabilitation practices, blister management, and wound care. Each author reviewed the tables for accuracy and contributed to the consensus manuscript.

**Results:**

The review demonstrated that over three quarters of the protocols addressed and reached consensus on performing rapid rewarming, administration of thrombolytics, the therapeutic window for thrombolytics, and therapeutic dosing of thrombolytics. However, variability remained in allowable treatment windows, rewarming temperature and process, and pain management during rewarming. Disagreement also occurred around the grades for thrombolytic treatment, thrombolytic contraindications and cautions, post thrombolytic anticoagulation and duration, and the role of therapy and splinting and appropriate wound care. The greatest variability was in contraindications, where several protocols excluded patients based on dementia, age, or ability to consent, while others lacked clarity regarding “recent” trauma. There were not enough centers using iloprost to draw any conclusions regarding consensus, although the centers that responded are largely following guidelines published by Poole et al.

**Conclusions:**

Centers with frostbite experience must share protocols, particularly as extreme cold events increasingly affect regions unaccustomed to such injuries. Current literature is inconsistent and often omits key details needed for meta-analysis. Harmonization of frostbite care protocols will support comparative effectiveness research and accelerate development of optimized, evidence-based treatments.

**Applicability of Research to Practice:**

This consensus manuscript will aid hospitals and emergency centers in developing site-specific protocols for the management of frostbite injuries.

**Funding for the study:**

The Department of Defense Military Burn Research Program.

